# Malignant Hypertension–Induced Thrombotic Microangiopathy Mimicking Atypical Hemolytic Uremic Syndrome and Thrombotic Thrombocytopenic Purpura

**DOI:** 10.1016/j.jaccas.2025.106031

**Published:** 2025-11-13

**Authors:** Mariana Valdez-Thomas, Michelle Carrasquel-Alvarez, Maria del Pilar Acosta, Ynsnardy Jose Hurtado-Leon, Mauricio Sosa Quintanilla, Sumesh Khanal

**Affiliations:** a Montefiore Medical Center at New Rochelle, Albert Einstein College of Medicine, Bronx, New York, USA; b McLaren Greater Lansing, Michigan State University, East Lansing, Michigan, USA

**Keywords:** blood tests, hemostasis, hypertension

## Abstract

**Background:**

Malignant hypertension can present with thrombotic microangiopathy (TMA) that closely resembles thrombotic thrombocytopenic purpura (TTP) and atypical hemolytic uremic syndrome (aHUS). Distinguishing these entities is critical because treatment strategies differ.

**Case Summary:**

We present a 26-year-old man with no prior medical history who developed abdominal pain, blurred vision, and severe hypertension (252/161 mm Hg). Initial management prioritized blood pressure control for malignant hypertension with acute kidney injury. Laboratory testing revealed anemia, thrombocytopenia, hemolysis, and renal dysfunction, raising concern for aHUS/TTP and prompting early nephrology consultation. Further evaluation, including preserved ADAMTS13 activity and the context of hypertensive retinopathy, supported malignant hypertension–induced TMA. With aggressive blood pressure control, renal function and platelet count improved, avoiding unnecessary plasma exchange.

**Discussion:**

TMA induced by malignant hypertension mimics TTP and aHUS but typically presents with extreme hypertension, hypertensive retinopathy, relatively higher platelet counts, preserved ADAMTS13 activity, and biopsy- or smear-proven vascular changes. Recognizing these distinctions and consulting nephrology early prevents unnecessary interventions and ensures appropriate therapy.

**Conclusions:**

Malignant hypertension is an important cause of secondary TMA. Controlled blood pressure reduction is the cornerstone of therapy. Multidisciplinary collaboration with nephrology helps confirm the diagnosis and guide plasma exchange decisions.

## Graphical Abstract

**Figure:**
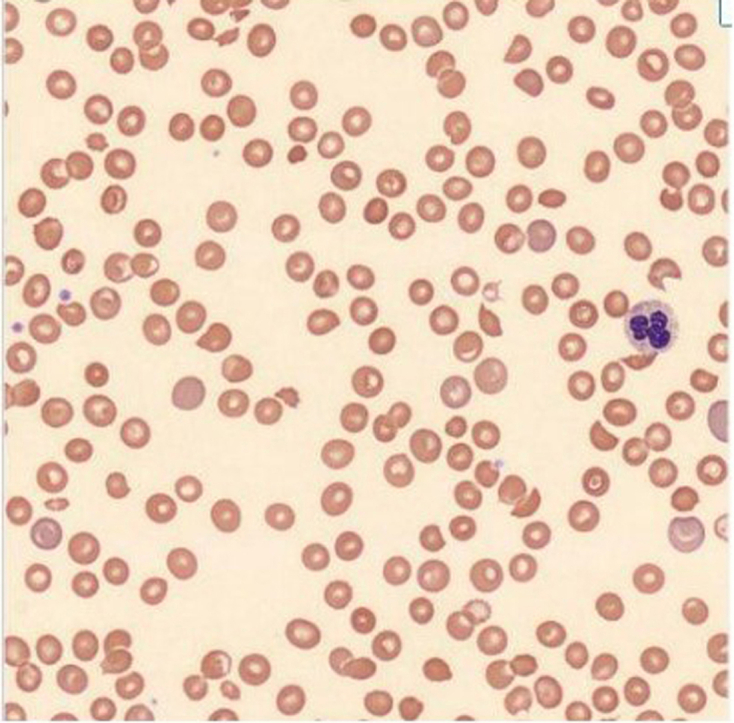

Thrombotic microangiopathies (TMAs) are a heterogeneous group of disorders characterized by microangiopathic hemolytic anemia (MAHA), thrombocytopenia, and end-organ injury. Primary syndromes include thrombotic thrombocytopenic purpura (TTP) and atypical hemolytic uremic syndrome (aHUS), whereas secondary forms occur in settings such as pregnancy, infection, drugs, and malignant hypertension.^1^, ^2^, ^3^ Malignant hypertension–associated TMA is under-recognized and often misdiagnosed as TTP/aHUS, leading to unnecessary plasma exchange.^1^^,^^4^ Here we describe a case of malignant hypertension–induced TMA, review its distinguishing features, and propose a practical teaching algorithm.

**Take-Home Messages**

• Malignant hypertension can present as a secondary thrombotic microangiopathy closely mimicking TTP and aHUS.
• Recognizing malignant hypertension–induced TMA early is crucial to avoid unnecessary plasma exchange and ensure timely, blood pressure–targeted therapy.

## Case Presentation

A 26-year-old man presented with 3 days of abdominal pain and vomiting. He reported blurred vision, early morning headaches, and fatigue. His blood pressure was 252/161 mm Hg, with heart rate 118 beats/min, respiratory rate 18 breaths/min, oxygen saturation 98% on room air, and a body mass index of 32.

### Investigations

**Figure 1:**
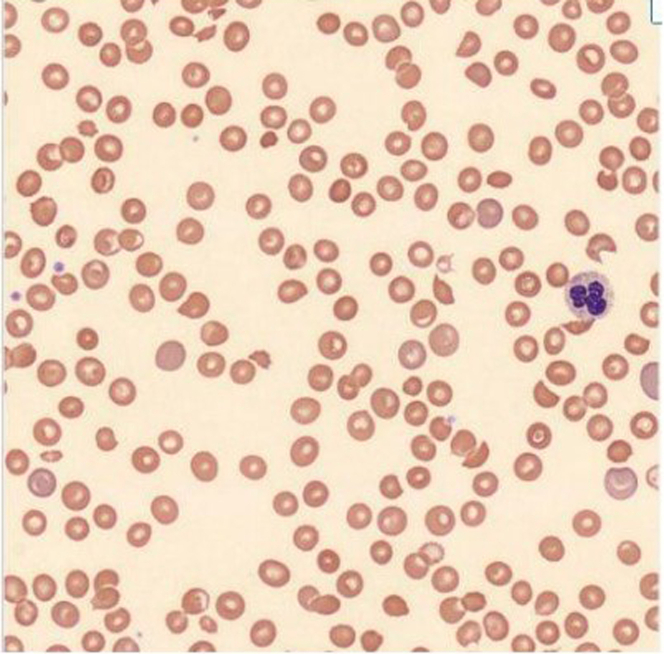
Peripheral Blood Smear (Wright-Giemsa Stain, 1,000×) Peripheral smear demonstrating multiple schistocytes and polychromasia, consistent with microangiopathic hemolytic anemia in the setting of thrombotic microangiopathy.

Examination revealed a distressed, obese male with right lower quadrant tenderness, no petechiae or rash, and no focal deficits. Laboratory tests demonstrated hemoglobin: 10.5 g/dL, platelets: 82 × 10^3^/μL, creatinine: 2.63 mg/dL, elevated lactate dehydrogenase and indirect bilirubin, modest troponin elevation, and schistocytes on smear (Figure 1, Table 1).

**Table 1:** Laboratory Results at Admission (Day 1)

Category	Key Results	Reference Range	Interpretation
Complete blood count	Hb: 10.5 g/dL (L), platelets: 82 × 10^3^/μL (L), WBC: 11.9 × 10^3^/μL (H)	Hb: 14-17.4, platelets: 130-400, WBC: 4.8-10.8	Anemia, thrombocytopenia, leukocytosis
Renal function	Creatinine: 2.63 mg/dL (H), BUN: 26 mg/dL (H), eGFR: 33	Creatinine: 0.5-1.2, BUN: 9-21	Acute kidney injury
Hemolysis panel	LDH: 1265 IU/L (H), total bilirubin: 2.0 mg/dL (H), haptoglobin: <10 mg/dL (L)	LDH: 0-240, total bilirubin: 0.2-1.2, haptoglobin: 30-200	Microangiopathic hemolysis
Liver enzymes	AST: 53 U/L (H), ALT: 17	AST: 10-42, ALT: 10-42	Mild AST elevation
Cardiac marker	Troponin I: 0.06 ng/mL (H)	<0.03	Demand ischemia/strain
Urinalysis	Proteinuria: 300 mg/dL, hematuria: 3+ (6 RBC/HPF), hyaline casts: 3/LPF	Proteinuria: <10, RBC: 0-3, casts: 0-1	Consistent with nephropathy/TMA

ALT = alanine aminotransferase; AST = aspartate aminotransferase; BUN = blood urea nitrogen; eGFR = estimated glomerular filtration rate; Hb = hemoglobin; HPF = high power field; LDH = lactate dehydrogenase; LPF = low power field; RBC = red blood cell; TMA = thrombotic microangiopathy; WBC = white blood cell.

**Figure 2:**
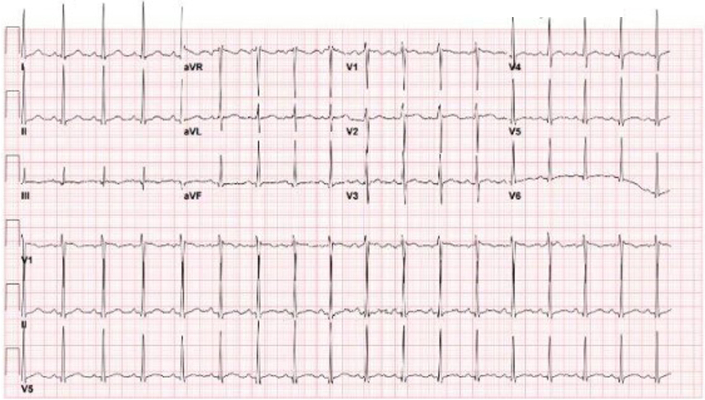
12-Lead Electrocardiogram Electrocardiogram on presentation showing sinus tachycardia (heart rate: 118 beats/min) with nonspecific ST-T abnormalities and voltage criteria suggestive of left ventricular hypertrophy, compatible with hypertensive cardiac strain.

Electrocardiogram showed sinus tachycardia with nonspecific ST-T abnormalities and voltage criteria for left ventricular hypertrophy (LVH), consistent with hypertensive cardiac strain (Figure 2).

**Figure 3:**
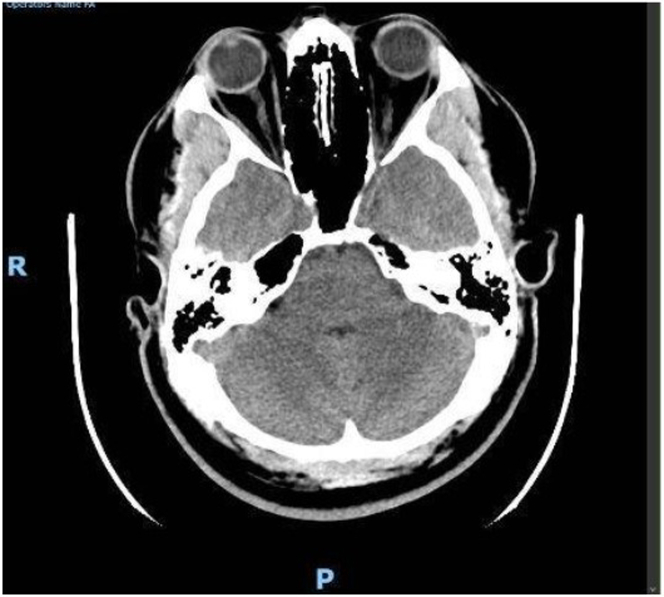
Head Computed Tomography (Noncontrast) Axial noncontrast computed tomography scan of the brain showing no acute intracranial hemorrhage, infarction, or mass effect.

**Figure 4:**
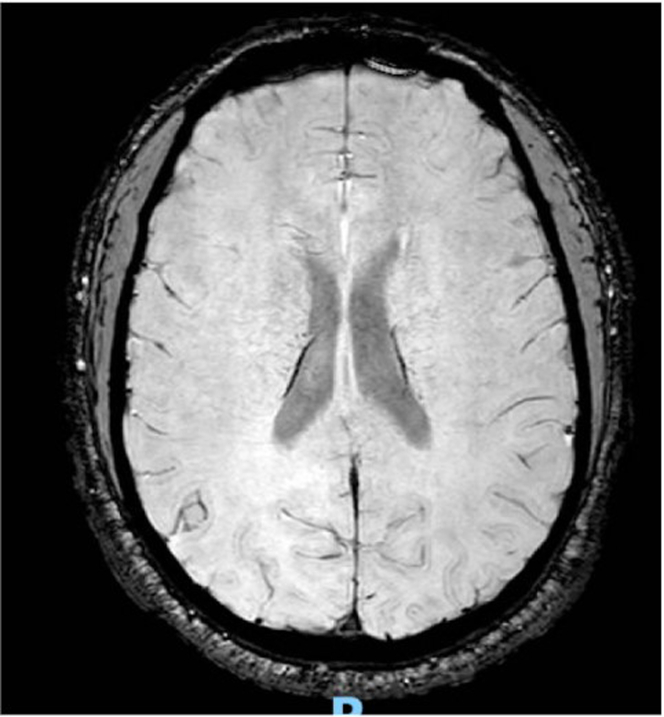
Brain MRI (T2-Weighted Axial Sequence) MRI of the brain demonstrating no evidence of acute infarction, hemorrhage, or posterior reversible encephalopathy syndrome (PRES). MRI = magnetic resonance imaging.

Computed tomography of the head showed no acute abnormality (Figure 3). Computed tomography of the abdomen revealed distal ileal edema with mesenteric adenopathy. Ophthalmology noted disc edema, cotton-wool spots, and hemorrhages consistent with malignant hypertensive retinopathy. Brain magnetic resonance imaging demonstrated no evidence of acute infarction, hemorrhage, or posterior reversible encephalopathy syndrome (Figure 4).

**Figure 5:**
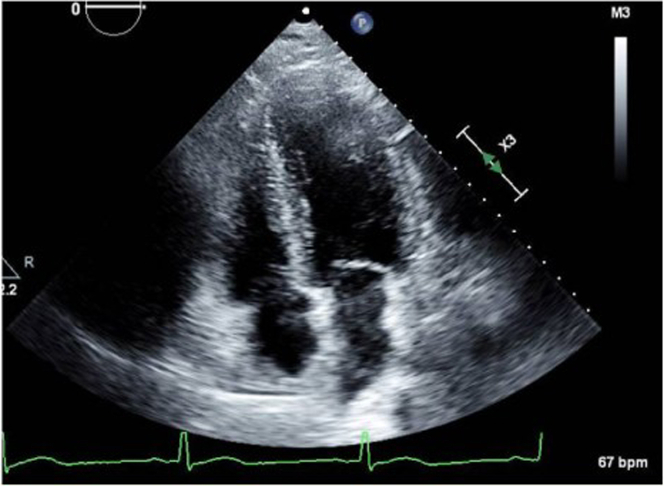
Transthoracic Echocardiography (Apical 4-Chamber View) Echocardiography demonstrating normal left ventricular size and systolic function, preserved right ventricular function, and no significant valvular stenosis or regurgitation.

Transthoracic echocardiography in the apical 4-chamber view revealed normal left ventricular size and systolic function, preserved right ventricular function, and no significant valvular stenosis or regurgitation (Figure 5).

### Management

On arrival, the priority was stabilization of the patient's malignant hypertension with acute kidney injury. Admission laboratory tests showed anemia, thrombocytopenia, hemolysis, and renal dysfunction (hemoglobin: 10.5 g/dL, platelets: 82 × 10^3^/μL, creatinine: 2.63 mg/dL, lactate dehydrogenase: 1265 IU/L, bilirubin: 2.0 mg/dL, haptoglobin: <10 mg/dL, proteinuria, and hematuria). These findings raised concern for aHUS/TTP, leading to early nephrology consultation. Further evaluation, including preserved ADAMTS13 activity and the context of severe hypertension with retinopathy, supported malignant hypertension–induced TMA, and plasma exchange was deferred.

## Discussion

### Pathophysiology and diagnostic challenges

Malignant hypertension is a life-threatening emergency characterized by severe blood pressure elevation and acute target organ damage. One of its most feared complications is TMA, which arises from endothelial injury, fibrinoid necrosis, and vascular narrowing, ultimately producing shear stress, MAHA, thrombocytopenia, and acute kidney injury.^1^^,^^2^^,^^5^ This clinical phenotype often overlaps with primary TMAs such as TTP and aHUS, making diagnosis challenging.^1^^,^^4^^,^^6^

Several features help differentiate malignant hypertension–induced TMA from primary TMAs. The presence of markedly elevated blood pressure with end-organ damage, such as hypertensive retinopathy or LVH, strongly favors malignant hypertension TMA.^1^^,^^2^^,^^5^ In contrast, TTP often presents with profound neurologic manifestations, whereas aHUS is frequently associated with gastrointestinal symptoms and progressive renal dysfunction.^6^

The severity of thrombocytopenia also offers diagnostic clues. Platelet counts in malignant hypertension–induced TMA are usually moderately reduced (>50 × 10^9^/L), whereas TTP typically presents with severe thrombocytopenia (<30 × 10^9^/L).^1^^,^^4^ ADAMTS13 activity remains preserved in malignant hypertension TMA, while a severe deficiency (<10%) is diagnostic of TTP.^4^^,^^7^ Complement abnormalities may be identified in a subset of patients with hypertension-associated TMA, suggesting that complement dysregulation contributes to pathogenesis in some cases^3^^,^^8^ (Table 2).

**Table 2:** Evolution of Blood Pressure and Key Laboratory Values

Time	SBP/DBP (mm Hg)	Platelets (×10^3^/μL)	Creatinine (mg/dL)	LDH (IU/L)	Total Bilirubin (mg/dL)
Day 1 (admission)	215/145	82	2.63	1,104	2
Day 3	189/125	106	2.68	990	1.1
Day 5	154/97	170	2.67	673	0.6
Discharge	129/64	427	2.58	218	0.3

DBP = diastolic blood pressure; LDH = lactate dehydrogenase; SBP = systolic blood pressure.

Histopathology is also valuable. In malignant hypertension TMA, renal biopsy demonstrates arteriolar and arterial changes with intimal edema, fibrin thrombi, and ischemic glomeruli, contrasting with the diffuse capillary thrombi seen in primary TMAs.^6^^,^^7^

### Therapeutic implications

Treatment approaches differ markedly and highlight the importance of multidisciplinary collaboration. In malignant hypertension TMA, aggressive yet controlled blood pressure reduction is the cornerstone of therapy.^2^^,^^5^^,^^7^ In contrast, TTP requires urgent plasma exchange,^4^^,^^9^ and aHUS requires complement inhibition.^8^^,11^ Because the clinical presentation can overlap, nephrology consultation should be obtained early whenever malignant hypertension–induced TMA is suspected but TTP/aHUS cannot be immediately excluded. Nephrology plays a central role in determining the need for plasma exchange and performing renal biopsy if diagnostic uncertainty persists. In our case, early cardiology involvement ensured blood pressure control, while nephrology assessment guided the decision to withhold plasma exchange once malignant hypertension TMA was confirmed. Early recognition of this distinction prevents unnecessary interventions and ensures that therapy is appropriately directed.

### Relevance in young adults

Although malignant hypertension is classically described in middle-aged adults, younger patients may also be affected. Epidemiologic studies suggest that <5% of malignant hypertension cases occur in individuals younger than 30 years.^1^^,^^2^^,^^5^ When malignant hypertension presents in this age group, secondary etiologies such as renal parenchymal disease, renovascular hypertension, or endocrine causes (eg, pheochromocytoma) are often implicated.^1^^,^^10^ The occurrence of malignant hypertension–induced TMA in a 26-year-old without known comorbidities, as in our case, is therefore unusual and clinically significant. Such presentations highlight the importance of maintaining a broad differential diagnosis in young patients with thrombotic microangiopathy and severe hypertension, as the correct recognition of malignant hypertension can prevent unnecessary plasma exchange and guide appropriate therapy with blood pressure control (Tables 3 and 4).^1^^,^^3^^,^^4^

**Table 3:** Differential Diagnosis of Thrombotic Microangiopathy Syndromes

Feature	Malignant HTN-TMA	TTP	aHUS
Blood pressure	Very high (often >180/120 mm Hg)	Usually normal/mild ↑	Variable
Fundoscopy	Hypertensive retinopathy (hemorrhages, cotton-wool spots, papilledema)	Rare	Rare
Platelets	↓ moderate (often >50 × 10^3^/μL)	↓ severe (<30 × 10^3^/μL)	↓ mild to moderate
Hemolysis laboratory tests	Schistocytes, ↑LDH, ↑bilirubin, ↓haptoglobin	Same	Same
Neurologic signs	Headache, blurred vision (due to HTN)	Prominent (confusion, seizures, stroke)	Possible but less frequent
Renal involvement	Acute kidney injury prominent	Mild to moderate renal failure	Severe renal failure typical
ADAMTS13	Normal or mildly ↓	Severely ↓ (<10%)	Normal
Complement	Normal (rare abnormalities)	Normal	Abnormal (↓C3, Factor H/I mutations, anti–Factor H antibodies)
Biopsy (if done)	Arteriolar changes, fibrinoid necrosis, ischemic glomeruli	Glomerular capillary thrombi	Diffuse glomerular thrombi
Response to therapy	Improves with blood pressure control	Requires plasma exchange	Requires complement inhibition (eculizumab)

aHUS = atypical hemolytic uremic syndrome; HTN = hypertension; LDH = lactate dehydrogenase; TMA = thrombotic microangiopathy; TTP = thrombotic thrombocytopenic purpura.

**Table 4:** Specialized Laboratory and Diagnostic Work-Up

Category	Test	Result	Interpretation
TTP exclusion	ADAMTS13 activity	Normal	TTP ruled out (not <10%)
Hemolysis type	Direct Coombs	Negative	Not autoimmune hemolysis
Complement	C3: 144 mg/dL (N), C4: 50 mg/dL (N), CH50: 58 U/mL (N)	Normal levels	aHUS less likely
Genetics	15-gene panel	No pathogenic variants	No monogenic aHUS
Genetics	Risk haplotypes	CFH-H3 heterozygous; MCP/CD46 negative	Risk allele only, not diagnostic
Genetics	CFHR1/CFHR3	Heterozygous deletions	Uncertain significance
Genetics	C5 variants	Negative	Sensitive to eculizumab if ever needed
Autoimmune causes	ANA, dsDNA, ANCA, anti-GBM	All negative	Excluded autoimmune TMAs
Coagulation	INR: 1.20, PT: 15.1 seconds (slight ↑), PTT: normal	No DIC	—
Renin-aldosterone	Renin: ↑ (8.06 → 1.1 ng/mL/h), aldosterone: 10-13 ng/dL	Secondary activation	Hypertension-driven
Renal imaging	Duplex ultrasound	No stenosis, normal flow	Excluded renovascular HTN
Renal biopsy	Arteriolar intimal edema, striped fibrosis, interstitial nephritis; no diffuse TMA	Consistent with malignant hypertension	—

aHUS = atypical hemolytic uremic syndrome; DBP = diastolic blood pressure; DIC = disseminated intravascular coagulation; HTN = hypertension; INR = international normalized ratio; LDH = lactate dehydrogenase; PT = prothrombin time; PTT = partial thromboplastin time; SBP = systolic blood pressure; TMA = thrombotic microangiopathy; TTP = thrombotic thrombocytopenic purpura.

### Teaching algorithm

Step 1: Identify TMA phenotype (MAHA + thrombocytopenia + acute kidney injury).

Step 2: Clinical stratification:

• Severe hypertension with retinopathy/LVH → malignant hypertension–induced TMA
• Profound thrombocytopenia or neurologic symptoms → TTP

Step 3: Laboratory testing: complete blood count/smear, hemolysis panel, creatinine/urinalysis, ADAMTS13 activity, complement studies.

Step 4: Immediate therapy:

• If TTP cannot be excluded → initiate plasma exchange.^3^^,^^6^
• If malignant hypertension is dominant → initiate blood pressure control.^1^^,^^2^^,^^5^

Step 5: Diagnostic confirmation: peripheral smear, renal biopsy (if performed), and complement testing.^4^^,^^7^^,^^8^^,^^10^

Step 6: Reassessment:

• Improvement with blood pressure control → malignant hypertension–induced TMA
• Lack of improvement → evaluate for overlapping etiologies

## Conclusions

Malignant hypertension is an under-recognized cause of secondary TMA that can closely mimic TTP and aHUS. At emergency presentation, blood pressure stabilization is essential, but the concurrent findings of microangiopathic hemolytic anemia and thrombocytopenia should prompt inclusion of TTP and aHUS in the differential. Nephrology consultation is recommended early in such cases to guide consideration of plasma exchange and renal biopsy. Distinguishing features—including severe hypertension with retinopathy, relatively higher platelet counts, preserved ADAMTS13 activity, and characteristic smear or biopsy findings—help direct appropriate therapy. In our case, multidisciplinary collaboration between the cardiology and nephrology teams allowed early recognition of malignant hypertension–induced TMA, prevented unnecessary plasma exchange, and led to recovery with blood pressure control alone.

## Funding Support and Author Disclosures

The authors have reported that they have no relationships relevant to the contents of this paper to disclose.

## References

[1] Khanal N., Dahal S., Upadhyay S., Bhatt V.R., Bierman P.J. (2015). Differentiating malignant hypertension–induced TMA from thrombotic thrombocytopenic purpura. Ther Adv Hematol.

[2] van den Born B.J.H., Honnebier U.P., Koopmans R.P., van Montfrans G.A. (2005). Microangiopathic hemolysis and renal failure in malignant hypertension. Hypertension.

[3] Masias C., Vasu S., Cataland S.R. (2017). None of the above: thrombotic microangiopathy beyond TTP. Blood.

[4] Mitaka C., Hirata Y., Uchida T., Wada H., Kumada Y. (2016). Malignant hypertension with thrombotic microangiopathy: a case report and review. Intern Med.

[5] Song J., Kim H., Jang H. (2024). Hypertensive emergency with thrombotic microangiopathy: a diagnostic challenge. J Clin Med.

[6] George J.N., Nester C.M. (2014). Syndromes of thrombotic microangiopathy. N Engl J Med.

[7] Shibagaki Y., Fujita T. (2005). TMA in malignant hypertension vs HUS/TTP. Hypertens Res.

[8] Bora F., Ozturk S., Yildirim A., Altunoren O. (2021). Malignant hypertension–induced thrombotic microangiopathy mimicking aHUS. Turk J Nephrol.

[9] Noris M., Remuzzi G. (2009). Atypical hemolytic–uremic syndrome. N Engl J Med.

[10] Timmermans S., Abdul-Hamid M.A., Vanderlocht J. (2017). Hypertension-associated TMA may present with complement abnormalities. Kidney Int.

